# Supplementary material for: Genome-Wide Identification of WOX Genes in Korean Pine and Analysis of Expression Patterns and Properties of Transcription Factors
Source: Biology (Basel). 2025 Apr 12;14(4):411. doi: 10.3390/biology14040411 (PMC12024698; doi:10.3390/biology14040411)
Supplement: Supplementary file 1 [file biology-14-00411-s001.zip › Table S1.pdf]

Table S1: The Ka/Ks ratios of duplication for *PkWOXs*

PkWOX gene 1	*PkWOX* gene 2	Ka	Ks	Ka/Ks
PkWOX1	PkWOX21	0.648005079	4.131281759	0.156853276
PkWOX1	PkWOX16	0.861589031	4.637338073	0.18579388
PkWOX1	PkWOX5	0.773955579	4.038635894	0.19163787
PkWOX1	PkWOX14	0.622026467	3.130790452	0.198680326
PkWOX1	PkWOX18	0.678057789	3.044111356	0.222744082
PkWOX10	PkWOX14	0.381988848	3.51621382	0.108636411
PkWOX10	PkWOX18	0.718248224	4.38915186	0.163641689
PkWOX10	PkWOX13	0.374595961	2.098833381	0.178478179
PkWOX10	PkWOX15	0.389480149	1.916005811	0.203277123
PkWOX10	PkWOX17	0.559378256	2.665771766	0.209837265
PkWOX10	PkWOX11	0.365333472	1.616500227	0.226002735
PkWOX10	PkWOX12	0.408541329	1.802307474	0.226676821
PkWOX11	PkWOX17	0.551421652	3.1782371	0.173499218
PkWOX11	PkWOX14	0.037454934	0.16090313	0.232779397
PkWOX11	PkWOX18	0.71421958	2.373743052	0.300883274
PkWOX11	PkWOX15	0.0685206	0.204016218	0.335858593
PkWOX11	PkWOX12	0.048767175	0.114629403	0.425433384
PkWOX11	PkWOX13	0.043986545	0.097965137	0.44900203
PkWOX12	PkWOX18	0.742574773	2.127568996	0.349025002
PkWOX12	PkWOX14	0.039120703	0.107995475	0.362243907
PkWOX12	PkWOX17	0.681134328	1.716147267	0.396897365
PkWOX12	PkWOX13	0.023997248	0.04596205	0.522110048
PkWOX12	PkWOX15	0.083320536	0.151264443	0.550826976
PkWOX13	PkWOX21	0.752459272	3.795500193	0.198250358
PkWOX13	PkWOX17	0.542546814	2.585819908	0.209816164
PkWOX13	PkWOX14	0.029748099	0.092300689	0.32229553
PkWOX13	PkWOX18	0.762614849	1.759927399	0.433321766
PkWOX13	PkWOX15	0.089708571	0.116461522	0.770285066
PkWOX14	PkWOX17	0.585528888	1.981082004	0.295560147
PkWOX14	PkWOX15	0.079253182	0.240214952	0.329926098
PkWOX14	PkWOX18	0.772921082	2.171312989	0.355969446
PkWOX15	PkWOX17	0.564001152	2.310544115	0.244098846
PkWOX15	PkWOX20	0.904446453	3.268172372	0.276743804
PkWOX15	PkWOX19	0.978808277	2.714562411	0.360576818
PkWOX16	PkWOX20	0.521629382	3.926367904	0.132852905
PkWOX16	PkWOX19	0.358864469	1.367089307	0.262502579
PkWOX17	PkWOX18	0.679134936	2.952567457	0.230015045
PkWOX17	PkWOX21	0.60644737	1.969971072	0.307845825
PkWOX18	PkWOX21	0.608882281	2.567645144	0.23713646
PkWOX19	PkWOX20	0.590844571	1.503562987	0.392962966
PkWOX2	PkWOX12	0.712114039	2.796352441	0.254658186

PkWOX gene 1	*PkWOX* gene 2	Ka	Ks	Ka/Ks
PkWOX2	PkWOX13	0.74558929	2.884152482	0.258512438
PkWOX2	PkWOX15	0.743920104	2.737632844	0.27173845
PkWOX2	PkWOX3	0.18056237	0.601010126	0.300431494
PkWOX2	PkWOX7	0.342816428	0.943035884	0.363524266
PkWOX2	PkWOX4	0.307594125	0.810164055	0.379668936
PkWOX2	PkWOX8	0.344891534	0.899105446	0.383594089
PkWOX2	PkWOX5	0.316799435	0.799437802	0.396277778
PkWOX2	PkWOX9	0.337335038	0.850946255	0.396423436
PkWOX2	PkWOX6	0.313949469	0.767541651	0.409032485
PkWOX3	PkWOX17	0.791041699	4.879338045	0.1621207
PkWOX3	PkWOX12	0.780902357	3.002834404	0.260055085
PkWOX3	PkWOX13	0.784315843	2.838490011	0.276314463
PkWOX3	PkWOX8	0.339791178	1.152159123	0.294916883
PkWOX3	PkWOX6	0.359646664	1.18202699	0.30426265
PkWOX3	PkWOX9	0.344192229	1.111746149	0.309596061
PkWOX3	PkWOX4	0.36330104	1.145692535	0.317101691
PkWOX3	PkWOX5	0.36084152	1.112151163	0.324453665
PkWOX3	PkWOX7	0.360759578	1.0407336	0.346639695
PkWOX3	PkWOX11	0.786194494	1.959508286	0.401220296
PkWOX4	PkWOX11	0.92313517	5.228047559	0.176573598
PkWOX4	PkWOX12	0.743629593	2.876199693	0.258545884
PkWOX4	PkWOX17	0.774540146	2.230346471	0.347273465
PkWOX4	PkWOX6	0.020446876	0.038505247	0.531015306
PkWOX4	PkWOX9	0.046512112	0.07919438	0.587315808
PkWOX4	PkWOX5	0.014092273	0.021780115	0.647024714
PkWOX4	PkWOX7	0.033436155	0.050064922	0.667855934
PkWOX4	PkWOX8	0.082848993	0.114077366	0.726252676
PkWOX5	PkWOX14	0.687808157	4.250561123	0.161815849
PkWOX5	PkWOX11	0.71533194	2.530268052	0.282709944
PkWOX5	PkWOX13	0.670127307	2.146737411	0.312160818
PkWOX5	PkWOX12	0.672372235	1.781050106	0.377514497
PkWOX5	PkWOX17	0.79959415	2.087894376	0.382966763
PkWOX5	PkWOX6	0.012526387	0.02722622	0.460085433
PkWOX5	PkWOX9	0.037010284	0.07202914	0.513823764
PkWOX5	PkWOX7	0.031835018	0.049926007	0.63764398
PkWOX5	PkWOX8	0.073419189	0.106990612	0.686220857
PkWOX6	PkWOX14	0.72369236	3.143339958	0.230230382
PkWOX6	PkWOX17	0.791745676	2.505640689	0.31598532
PkWOX6	PkWOX13	0.718740936	2.266643051	0.317094893
PkWOX6	PkWOX11	0.839043165	2.294191151	0.365725046
PkWOX6	PkWOX12	0.832658568	2.065037807	0.403217106
PkWOX6	PkWOX9	0.069543187	0.154164765	0.451096507
PkWOX6	PkWOX7	0.036738779	0.067271957	0.546123243

PkWOX gene 1	*PkWOX* gene 2	Ka	Ks	Ka/Ks
PkWOX6	PkWOX8	0.075964877	0.13877487	0.54739649
PkWOX7	PkWOX15	0.883831418	3.349431089	0.263875086
PkWOX7	PkWOX14	0.829764199	2.801265458	0.296210485
PkWOX7	PkWOX11	0.905633975	2.761307974	0.327972824
PkWOX7	PkWOX13	0.813411722	2.259090637	0.36006157
PkWOX7	PkWOX12	0.755833746	2.023101069	0.373601575
PkWOX7	PkWOX9	0.035278781	0.093246314	0.378339681
PkWOX7	PkWOX8	0.072171024	0.133544108	0.540428366
PkWOX8	PkWOX19	0.892611764	3.735132065	0.238977297
PkWOX8	PkWOX11	0.700397528	2.816372082	0.248687854
PkWOX8	PkWOX14	0.763520056	1.986043718	0.384442724
PkWOX8	PkWOX17	0.796136702	2.015961766	0.394916568
PkWOX8	PkWOX12	0.706916754	1.622884016	0.435592899
PkWOX8	PkWOX13	0.737471787	1.598359123	0.461393047
PkWOX8	PkWOX15	0.827515258	1.744219406	0.474433007
PkWOX8	PkWOX9	0.005096282	0.005758186	0.885050021
PkWOX9	PkWOX14	0.770060796	1.986043718	0.387736075
PkWOX9	PkWOX17	0.854970784	2.150796961	0.39751348
PkWOX9	PkWOX12	0.712989662	1.622884016	0.439334946
PkWOX9	PkWOX13	0.743681931	1.598359123	0.465278372
PkWOX9	PkWOX15	0.873376286	1.401847015	0.623018259
