# Supplementary material for: Genome-Wide Identification of WOX Genes in Korean Pine and Analysis of Expression Patterns and Properties of Transcription Factors
Source: Biology (Basel). 2025 Apr 12;14(4):411. doi: 10.3390/biology14040411 (PMC12024698; doi:10.3390/biology14040411)
Supplement: Supplementary file 1 [file biology-14-00411-s001.zip › Table S3.pdf]

Table S3 Description of the interacting proteins

Gene ID	Description
Pkor02G01182	Transcription factor PRE6
Pkor02G01347	calmodulin-binding transcription activator 3 isoform X4 [*Amborella trichopoda*]
Pkor02G01783	expansin [*Pinus taeda*]
Pkor02G02392	Protein RKD5
Pkor02G02493	Ethylene-responsive transcription
Pkor04G01824	Mediator of RNA polymerase II
Pkor05G00426	hypothetical protein AXG93_2318s1400 [*Marchantia polymorpha subsp. ruderalis*]
Pkor05G01442	unknown [*Picea sitchensis*]
Pkor06G00136	unknown [*Picea sitchensis*]
Pkor06G00732	Mannan endo-1,4-beta-mannosidase
Pkor08G02153	Nuclear transcription factor Y subunit
Pkor10G02164	Subtilisin-like protease SBT2.4
Pkor10G02302	F-box/LRR-repeat protein 17
Pkor11G00013	receptor protein kinase-like protein ZAR1 isoform X2 [*Amborella trichopoda*]
Pkor11G01640	Homeobox-leucine zipper protein HOX32
Pkor12G00784	CKX5 [*Pinus tabuliformis*]
Pkor12G00797	hypothetical protein JHK86_031239 [*Glycine max*]
