# Supplementary material for: Genome-Wide Identification of WOX Genes in Korean Pine and Analysis of Expression Patterns and Properties of Transcription Factors
Source: Biology (Basel). 2025 Apr 12;14(4):411. doi: 10.3390/biology14040411 (PMC12024698; doi:10.3390/biology14040411)
Supplement: Supplementary file 1 [file biology-14-00411-s001.zip › Table S4.pdf]

Table S4 The Primer List of PkWOXs for gene cloning, analysis of transcriptional activation activity and subcellular localization

引物名称<br>Primer name	引物序列（5'-3' ）<br>Primers sequence （5'-3' ）
WOX2-F	ATGAATCCCAACAAGCATTG
WOX2-R	TCATATCACGTAATACATAGCTCCAT
WOX3-F	ATGAATCCTGGCAAGCATTG
WOX3-R	TCATATCAAGTAATATATAGCTCCATG
WOX16-F	ATGGCCAACCCCAATTTCGTGGCTTTGC
WOX16-R	TTAAGTTCCCCGATATGGGTC
pFGC-WOX2-F	GCATGGACGAGCTGTACAAGGGATCCATGAATCCCAACAAGCATTG
pFGC-WOX2-R	ATTAACTCTCTAGACTCACCTAGGATCCTCATATCACGTAATACATAGCTCCAT
pFGC-WOX3-F	GCATGGACGAGCTGTACAAGGGATCCATGAATCCTGGCAAGCATTG
pFGC-WOX3-R	ATTAACTCTCTAGACTCACCTAGGATCCTCATATCAAGTAATATATAGCTCCATG
pFGC-WOX16-F	GCATGGACGAGCTGTACAAGGGATCCATGGCCAACCCCAATTTCGTGGCTTTGC
pFGC-WOX16-R	ATTAACTCTCTAGACTCACCTAGGATCCTTAAGTTCCCCGATATGGGTC
pGBKT7- WOX2-F	GCATATGGCCATGGAGGCCGAATTCATGAATCCCAACAAGCATTG
pGBKT7- WOX2-R	CGGCCGCTGCAGGTCGACGGATCCTCATATCACGTAATACATAGCTCCAT
pGBKT7- WOX3-F	GCATATGGCCATGGAGGCCGAATTCATGAATCCTGGCAAGCATTG
pGBKT7- WOX3-R	CGGCCGCTGCAGGTCGACGGATCCTCATATCAAGTAATATATAGCTCCATG
pGBKT7-WOX16-F	GCATATGGCCATGGAGGCCGAATTCATGGCCAACCCCAATTTCGTGGCTTTGC
pGBKT7- WOX16-R	CGGCCGCTGCAGGTCGACGGATCCTTAAGTTCCCCGATATGGGTC
