# Supplementary figures and images for: Genome-Wide Identification of WOX Genes in Korean Pine and Analysis of Expression Patterns and Properties of Transcription Factors
Source: Biology (Basel). 2025 Apr 12;14(4):411. doi: 10.3390/biology14040411 (PMC12024698; doi:10.3390/biology14040411)

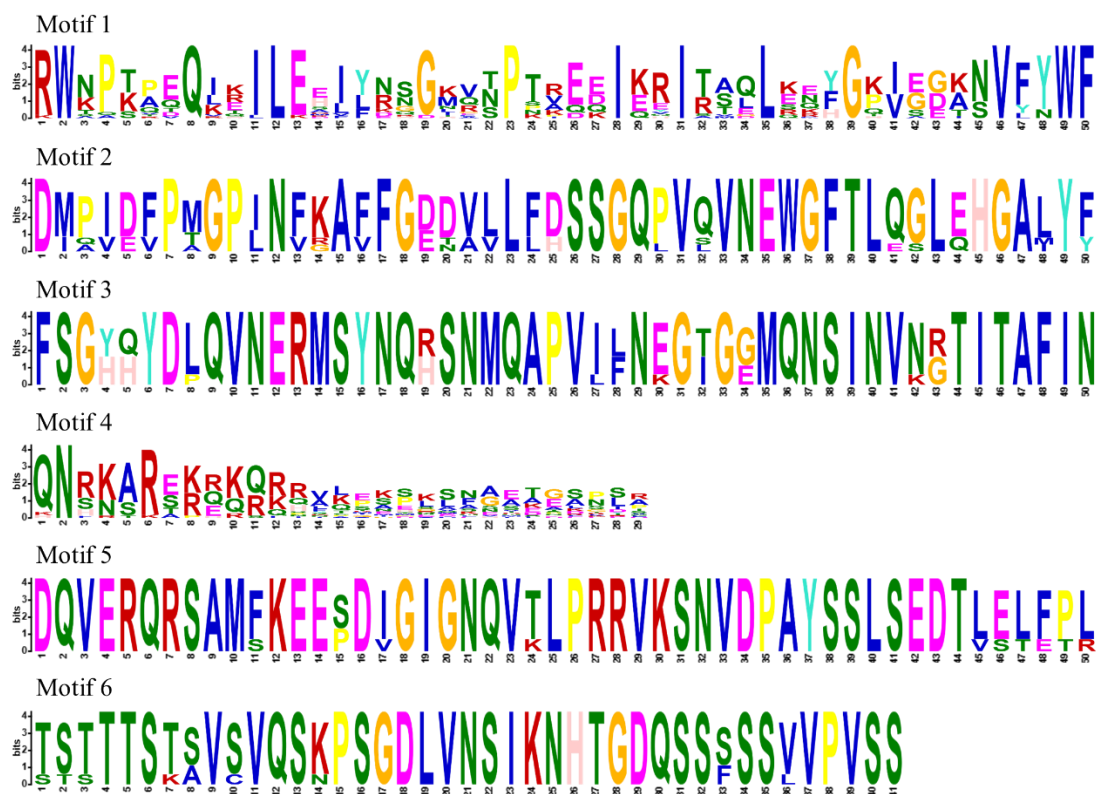

Motif 7

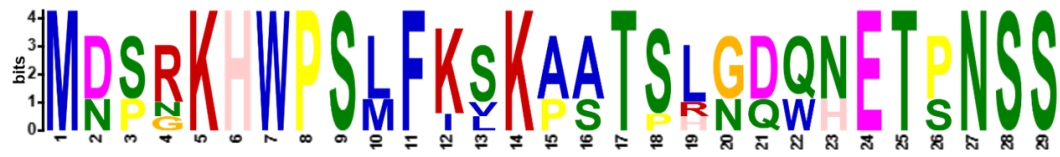

Motif 8

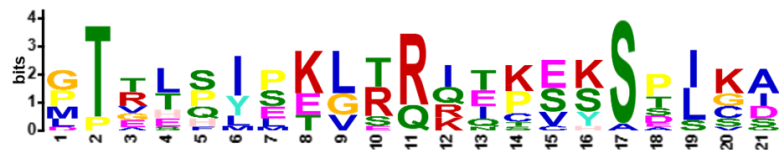

Motif 9

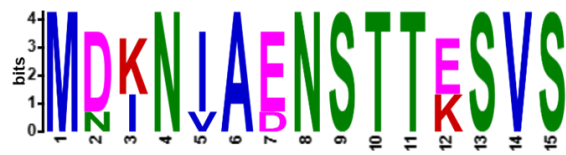

Motif 10

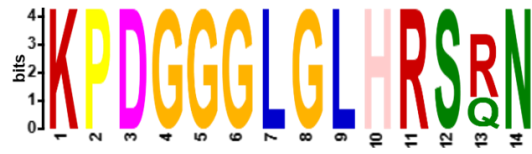

Motif 11

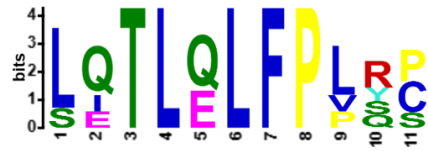

Supplement: Supplementary file 1 [file biology-14-00411-s001.zip › Figure S1.pdf]
